# Unraveling the mystery: How autophagy deficiency in dopaminergic neurons drives human Parkinson’s disease

**DOI:** 10.1186/s13041-025-01235-5

**Published:** 2025-07-24

**Authors:** Sachiko Noda, Nobutaka Hattori

**Affiliations:** 1 Department of Neurology, Juntendo University Graduate School of Medicine, 2-1-1 Hongo, Bunkyo-ku, Tokyo, 113-8421 Japan; 2 Neuron-Glia Crosstalk centerï Juntendo, Juntendo University Graduate School of Medicine, 2-1-1 Hongo, Bunkyo-ku, Tokyo, 113-8421 Japan; 3 Neurodegenerative Disorders Collaborative Laboratory, RIKEN Center for, Brain Science 2-1 Hirosawa, Wako, 351-0198 Saitama Japan

**Keywords:** Parkinson’s disease, Α-synuclein, Autophagy, Dopaminergic neurons

## Abstract

Alpha-synuclein (α-synuclein), a key component of Lewy body pathology, is a classical hallmark of Parkinson’s disease. In previous studies, our group has examined dopaminergic neuron-specific *Atg7* autophagy-deficient mice, observing α-synuclein aggregation in vivo. This pathological process led to dopamine neuron loss and age-related motor impairments. Further, in a recent study, we developed a new mouse model by crossing human α-synuclein bacterial artificial chromosome transgenic mice with dopaminergic neuron-specific *Atg7* conditional knockout mice to further investigate these mechanisms. These model mice exhibited accelerated Lewy body-like pathology and motor dysfunction, providing additional evidence that autophagy deficiency exacerbates synuclein toxicity in vivo. This nano-review provides essential clues that autophagy deficiency in dopamine neurons may contribute to the onset of human synuclein diseases.

Parkinson’s disease (PD) is a neurodegenerative disorder characterized by the progressive loss of dopaminergic neurons in the substantia nigra pars compacta, combined with the accumulation of intracellular Lewy bodies containing misfolded α-synuclein and ubiquitin [1]. Malfunctioning of the ubiquitin-proteasome system (UPS) and the autophagy-lysosomal pathway (ALP) has been implicated in the buildup of protein aggregates, a pathological feature of PD. Autophagy is an essential cellular process that triggers the degradation of cytoplasmic proteins, damaged organelles, and other cellular components. This process involves the formation of double-membraned autophagosomes, which later fuse with lysosomes to facilitate degradation [2]. Significantly, the clearance of oligomeric synuclein relies heavily on the functionality of the autophagy-lysosome system [3–5]. Research using mice with brain-specific *Atg7* deletion has further highlighted the critical role of autophagy in the clearance of misfolded and aggregated proteins, reducing their accumulation, and associated neurodegeneration [6]. Acting as a selective autophagy receptor, p62 binds to both ubiquitin and LC3 to mediate the clearance of ubiquitinated proteins through autophagic degradation [4]. Notably, p62 has been identified in neuronal inclusions associated with various neurodegenerative disorders [7].

**Fig. 1:**
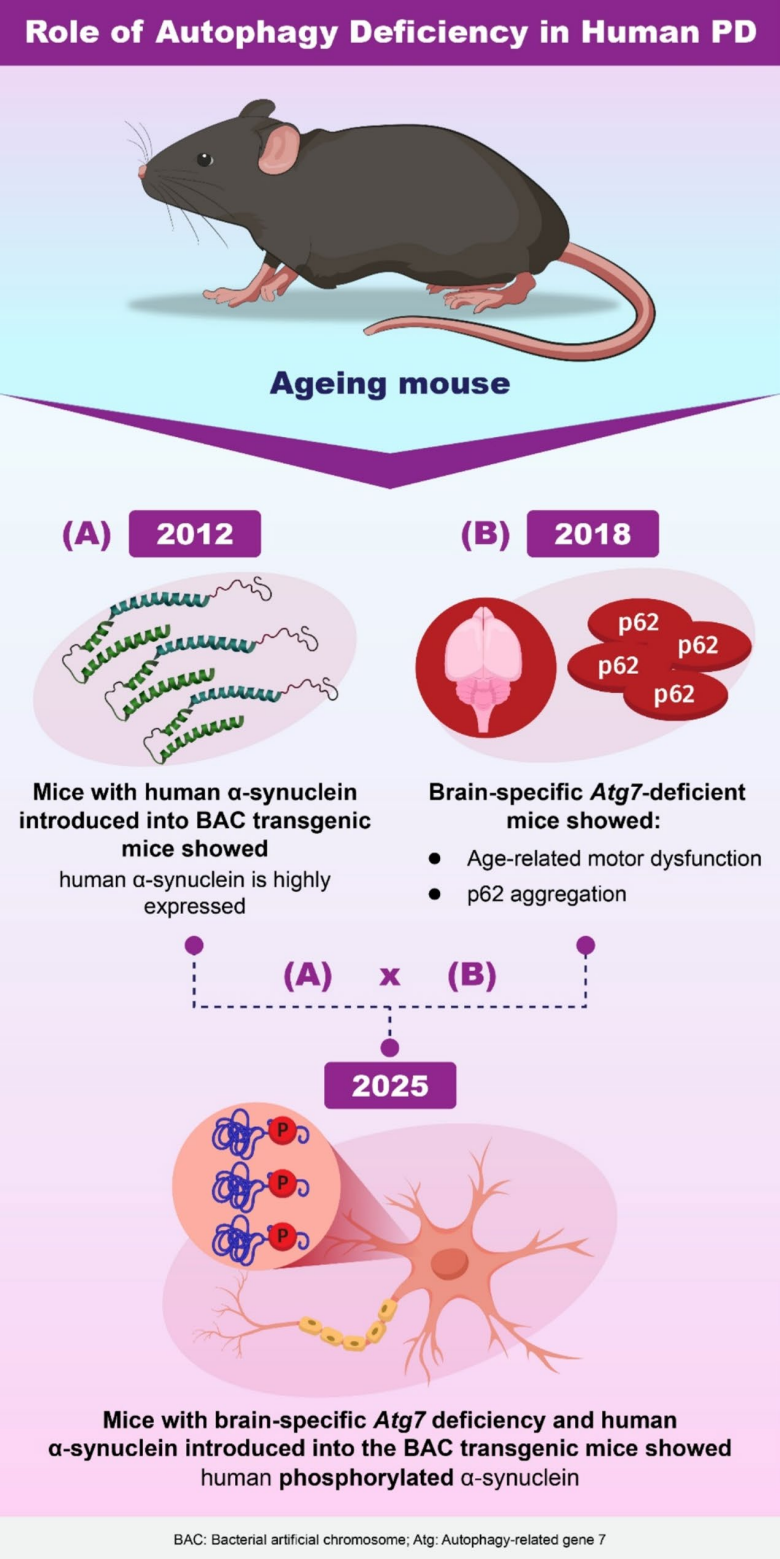
**A**) In 2012, BAC transgenic mice with human α-synuclein introduced were reported, showing increased expression levels of human α-synuclein. **B**) In 2018, brain-specific *Atg7*-deficient mice were reported to exhibit age-related motor dysfunction and p62 aggregation. **C**) In 2025, a crossbreed of mice (**A**) and (**B**), combining brain-specific *Atg7* deficiency and human α-synuclein introduced via BAC transgenics, demonstrated the presence of human phosphorylated α-synuclein in the brain

Numerous studies have employed α-synuclein transgenic rodent models to investigate the mechanisms driving human synucleinopathies [8]. These models have consistently demonstrated a link between elevated α-synuclein expression and toxic gain-of-function effects, resulting in earlier disease onset and more severe symptoms. In human PD, α-synuclein gene duplication or triplication leads to increases in the levels of RNA and protein associated with early onset and rapid progression. Similarly, transgenic animals with increased α-synuclein expression develop insoluble aggregates and inclusions, mimicking the pathology observed in human PD. These results indicate that selective autophagy impairment in the dopaminergic system exacerbates α-synuclein-related pathology.

Brain-specific *Atg7* deletion mice display age-related neuronal death [9]. Further, the addition of human α-synuclein to BAC tg mice [10] resulted in more pronounced motor dysfunction and neuronal loss, indicating the heightened toxicity of phosphorylated human α-synuclein. Recent studies have shown that prolonged autophagy disruption, combined with human α-synuclein expression, leads to widespread accumulation of phosphorylated α-synuclein in dopaminergic neurons [11] (Fig. 1).

Overall, these observations underscore the essential role of autophagy in α-synuclein degradation within dopaminergic neurons and highlight the importance of human α-synuclein in PD pathology. Protein degradation pathways, including autophagy, regulate α-synuclein clearance [3]. Disruptions in autophagy have been associated with the development of α-synuclein inclusions [5]. Additionally, α-synuclein overexpression has been shown to interfere with autophagic processes, indicating a bidirectional interaction between these mechanisms [12]. Furthermore, Lewy body-like aggregates resist degradation and can further compromise autophagic pathways [13]. Collectively, these findings suggest that the accumulation of α-synuclein may arise from a dynamic interplay between autophagy impairment and the formation of inclusions.

## Data Availability

No datasets were generated or analysed during the current study.

## Abbreviations

α-synuclein: Alpha-synuclein
PD: Parkinson’s disease
UPS: Ubiquitin-proteasome system
ALP: Autophagy–lysosomal pathway
tg: Transgenic
BAC: Bacterial artificial chromosome
TH: Tyrosine hydroxylase
